# Supplementary material for: OpenMM 7: Rapid development of high performance algorithms for molecular dynamics
Source: PLoS Comput Biol. 2017 Jul 26;13(7):e1005659. doi: 10.1371/journal.pcbi.1005659 (PMC5549999; doi:10.1371/journal.pcbi.1005659)
Supplement: S1 Source Code — (ZIP) [file pcbi.1005659.s002.zip › OpenMM-7.0.1-Source/docs/api-python/generated/simtk.openmm.app.pdbfile.PDBFile.html]

PDBFile — OpenMM 7.0.1.dev-5e86c4f documentation


# PDBFile¶

*class* `simtk.openmm.app.pdbfile.``PDBFile`(*file*, *extraParticleIdentifier='EP'*)¶
:   PDBFile parses a Protein Data Bank (PDB) file and constructs a Topology and a set of atom positions from it.

    This class also provides methods for creating PDB files. To write a file containing a single model, call
    writeFile(). You also can create files that contain multiple models. To do this, first call writeHeader(),
    then writeModel() once for each model in the file, and finally writeFooter() to complete the file.

    `__init__`(*file*, *extraParticleIdentifier='EP'*)¶
    :   Load a PDB file.

        The atom positions and Topology can be retrieved by calling getPositions() and getTopology().

        |  |  |
        | --- | --- |
        | Parameters: | - **file** (*string*) – the name of the file to load - **extraParticleIdentifier** (*string=’EP’*) – if this value appears in the element column for an ATOM record, the Atom’s element will be set to None to mark it as an extra particle |

    Methods

    |  |  |
    | --- | --- |
    | `__init__`(file[, extraParticleIdentifier]) | Load a PDB file. |
    | `getNumFrames`() | Get the number of frames stored in the file. |
    | `getPositions`([asNumpy, frame]) | Get the atomic positions. |
    | `getTopology`() | Get the Topology of the model. |
    | `writeFile`(topology, positions[, file, ...]) | Write a PDB file containing a single model. |
    | `writeFooter`(topology[, file]) | Write out the footer for a PDB file. |
    | `writeHeader`(topology[, file]) | Write out the header for a PDB file. |
    | `writeModel`(topology, positions[, file, ...]) | Write out a model to a PDB file. |

    `__delattr__`¶
    :   x.\_\_delattr\_\_(‘name’) <==> del x.name

    `__format__`()¶
    :   default object formatter

    `__getattribute__`¶
    :   x.\_\_getattribute\_\_(‘name’) <==> x.name

    `__hash__`¶

    `__reduce__`()¶
    :   helper for pickle

    `__reduce_ex__`()¶
    :   helper for pickle

    `__repr__`¶

    `__setattr__`¶
    :   x.\_\_setattr\_\_(‘name’, value) <==> x.name = value

    `__sizeof__`() → int¶
    :   size of object in memory, in bytes

    `__str__`¶

    `getNumFrames`()¶
    :   Get the number of frames stored in the file.

    `getPositions`(*asNumpy=False*, *frame=0*)¶
    :   Get the atomic positions.

        |  |  |
        | --- | --- |
        | Parameters: | - **asNumpy** (*boolean=False*) – if true, the values are returned as a numpy array instead of a list   of Vec3s - **frame** (*int=0*) – the index of the frame for which to get positions |

    `getTopology`()¶
    :   Get the Topology of the model.

    *static* `writeFile`(*topology*, *positions*, *file=<open file '<stdout>'*, *mode 'w'>*, *keepIds=False*, *extraParticleIdentifier=' '*)¶
    :   Write a PDB file containing a single model.

        |  |  |
        | --- | --- |
        | Parameters: | - **topology** (*Topology*) – The Topology defining the model to write - **positions** (*list*) – The list of atomic positions to write - **file** (*file=stdout*) – A file to write to - **keepIds** (*bool=False*) – If True, keep the residue and chain IDs specified in the Topology   rather than generating new ones. Warning: It is up to the caller to   make sure these are valid IDs that satisfy the requirements of the   PDB format. Otherwise, the output file will be invalid. - **extraParticleIdentifier** (*string=’ ‘*) – String to write in the element column of the ATOM records for atoms whose element is None (extra particles) |

    *static* `writeFooter`(*topology*, *file=<open file '<stdout>'*, *mode 'w'>*)¶
    :   Write out the footer for a PDB file.

        |  |  |
        | --- | --- |
        | Parameters: | - **topology** (*Topology*) – The Topology defining the molecular system being written - **file** (*file=stdout*) – A file to write the file to |

    *static* `writeHeader`(*topology*, *file=<open file '<stdout>'*, *mode 'w'>*)¶
    :   Write out the header for a PDB file.

        |  |  |
        | --- | --- |
        | Parameters: | - **topology** (*Topology*) – The Topology defining the molecular system being written - **file** (*file=stdout*) – A file to write the file to |

    *static* `writeModel`(*topology*, *positions*, *file=<open file '<stdout>'*, *mode 'w'>*, *modelIndex=None*, *keepIds=False*, *extraParticleIdentifier=' '*)¶
    :   Write out a model to a PDB file.

        |  |  |
        | --- | --- |
        | Parameters: | - **topology** (*Topology*) – The Topology defining the model to write - **positions** (*list*) – The list of atomic positions to write - **file** (*file=stdout*) – A file to write the model to - **modelIndex** (*int=None*) – If not None, the model will be surrounded by MODEL/ENDMDL records   with this index - **keepIds** (*bool=False*) – If True, keep the residue and chain IDs specified in the Topology   rather than generating new ones. Warning: It is up to the caller to   make sure these are valid IDs that satisfy the requirements of the   PDB format. Otherwise, the output file will be invalid. - **extraParticleIdentifier** (*string=’ ‘*) – String to write in the element column of the ATOM records for atoms whose element is None (extra particles) |

High performance molecular simulation on GPUs


### Navigation

- Application Layer
  - Loaders and Setup
  - Representation and Manipulation
  - Simulation
  - Reporting Output
  - Extras
- Library Layer

©2015, Stanford University and the Authors.
|
Powered by Sphinx 1.3.1
& Alabaster 0.7.7
|
Page source
